# Smoke-induced SAV1 Gene Promoter Hypermethylation Disrupts YAP Negative Feedback and Promotes Malignant Progression of Non-small Cell Lung Cancer

**DOI:** 10.7150/ijbs.73428

**Published:** 2022-07-11

**Authors:** Ting Liu, Wei Guo, Kai Luo, Lei Li, Jing Dong, Meijun Liu, Xingyuan Shi, Zhiyuan Wang, Jianlei Zhang, Jiang Yin, Ni Qiu, Minying Lu, Danyang Chen, Xiaoting Jia, Hao Liu, Yixue Gu, Yan Xiong, Guopei Zheng, Gang Xu, Zhimin He, Zhijie Zhang

**Affiliations:** 1 Affiliated Cancer Hospital and Institute of Guangzhou Medical University, Guangzhou Key Laboratory of “Translational Medicine on Malignant Tumor Treatment”, Guangzhou city, Guangdong, P. R. China.; 2 Department of Central Laboratory, The Fifth Affiliated Hospital, Guangzhou Medical University, Guangzhou, Guangdong, P. R. China.; 3 The State Key Laboratory of Respiratory, Guangzhou, Guangdong, P. R. China.

**Keywords:** SAV1, YAP, Lung Cancer, Methylation, Tobacco smoking

## Abstract

YAP (gene symbol *YAP1*) as a potential oncoprotein, is positively correlated with the malignancy of various tumors. However, overexpression of YAP alone in multiple normal tissue cells has failed to induce tumor formation and the underlying mechanism is poorly understood. Herein, we show that YAP activation directly induces transcription of its negative regulator, SAV1, to constitute a negative feedback loop, which plays a vital role in maintaining lung epithelial cell homeostasis and was dysregulated in non-small cell lung cancer (NSCLC). Notably, smoking promotes the hypermethylation of the *SAV1* promoter region, which disrupts YAP negative feedback by inactivating the Hippo pathway. Besides, exogenous overexpression of SAV1 can act as a traffic protein, activating the Hippo signaling and concurrently inhibiting the WNT pathway to decrease cancer cell growth. Furthermore, using the lung cancer organoids, we found that lentivirus-mediated SAV1 gene transfer combined with methylation inhibitor and YAP-TEAD inhibitor is a potential feasible clinical medication regimen for the lung cancer patient, especially among the smoking population. Thus, this SAV1 mediated feedback loop provides an efficient mechanism to establish the robustness and homeostasis of YAP regulation and as a potential target of gene therapy for the smoking NSCLC population.

## Introduction

Lung cancer causes more deaths than any other cancer across the globe 1. Besides, more than 80% of these deaths are attributed to tobacco smoking 2. Notably, lung cancer comprises non-small cell lung cancer (NSCLC) and small cell lung cancer (SCLC). NSCLC, accounting for up to 85% of all lung cancer types, can be primarily subdivided into two groups, including lung adenocarcinoma (LUAD) and lung squamous cell carcinoma (LUSC). Over the past years, significant progress in research and understanding of the NSCLC biology and management has been reported, consequently enabling the development of targeted therapies and immunotherapies that revolutionized lung cancer treatments 3, 4. The use of small-molecule tyrosine kinase inhibitors (TKI) significantly improves the prognosis of NSCLC patients. However, acquired resistance inevitably develops within 12 months, moreover, TKI-targeted therapies are only limited to non-LUSC histological subtypes with operable driver mutations 5. Immune checkpoint inhibitors (ICIs) targeting the programmed cell death-1 (PD-1)/programmed cell death-ligand 1 (PD-L1) pathway are the standard therapeutic options for cancer patients. However, many previous reports have revealed that a minority of patients with non-small-cell lung cancer (NSCLC) responds to ICIs in the clinical setting and alternative approaches are urgently needed to improve patient outcome 6.

Tobacco smoking causes lung cancer and promotes its development and this has been attributed to more than 60 carcinogens in cigarettes. These carcinogens directly mutate and trigger epigenetic changes in DNA, as well as indirect effects including infections, inflammation, and immunosuppression 7. The genomic pattern of lung cancer is significantly different among never-smokers and smokers. For instance, never-smokers exhibit a major cytosine to thymine transition with more actionable genetic alterations including EGFR mutations or ALK/ROS1 translocations, thus enabling targeted treatment. On the other hand, smokers have a significantly higher mutation frequency, primarily due to the conversion of cytosine to adenine nucleotides with molecular alterations like KRAS and TP53 remaining non-druggable 8-10.

Several studies have recently identified strong epigenetic signals related to tobacco smoking 11. DNA methylation, one of the major epigenetic mechanisms that causes silencing of gene expression that can be reversed by DNA-demethylating agents, regulate smoking-induced diseases including NSCLC 12. As a reversible process, DNA methylation is catalyzed by DNA methyltransferases (DNMTs) on the 5^th^ carbon of cytosine residues at CpG dinucleotides to form 5-methylcytosine (5mC), where gene promoter methylation usually exerts a repressive action on transcription 13. Unlike genetic changes, epigenetic changes including DNA methylation are potentially reversible, hence a strong therapeutic target candidate 14.

Originally identified in *Drosophila melanogaster*, the Hippo signaling pathway is an evolutionarily conserved regulator of organ size related to tumorigenesis 15, 16. In humans, the core kinase cascade of the Hippo pathway comprises Sterile-20 family kinases MST1/2 (Hippo in *Drosophila*), NDR family kinases LATS1/2 (Wts in *Drosophila*), the scaffolding protein SAV1 (Sav in *Drosophila*), and the adaptor protein MOB1 (Mats in *Drosophila*). Mechanically, activated MST1/2 kinase associates with its scaffolding partners SAV1 and phosphorylate LATS1/2, causing a LATS1/2 activation. The activated LATS1/2 kinase associate with MOB1 then advances to phosphorylate YAP on the phosphorylation site, causing inactivation of YAP by cytoplasmic sequestering and degradation. When the SAV1-MST1/2-LATS1/2 cascade axis is off, YAP translocates into the nucleus, interacts with the transcription enhancer (TEF) family of transcription factors (TEAD), and acts as a co-transcription factor. Abnormal hyperactivation of YAP/TAZ triggers overgrowth of tissues with major cancer characteristics including epithelial-mesenchymal transition (EMT), increased migration as well as potential for metastasis, therapeutic resistance, and cancer stem cell properties 17-19. Of note, activated YAP functions as a driver oncogene in multiple cancer types. Elevated nuclear levels of YAP and TAZ (YAP/TAZ) are associated with widespread aggressive cancers. Nevertheless, YAP overexpression in a few tissues does not induce tumor formation 20-22.

Negative feedback loops play vital roles in maintaining tissue homeostasis 23, 24. Yorkie/YAP induces its negative regulators including expanded (ex) and Merlin (Mer) in Drosophila and Lats2 or NF2 in mammalian cultured cells, thereby forming a negative feedback loop that participates in the regulation of the Hippo pathway and maintain organ homeostasis 25, 26. However, the biological significance of this potential negative feedback regulation remains unclarified. Additionally, despite the Hippo pathway being evolutionary conservative, many paralogous genes from different species make the pathway more complex and volatile.

This work discovered a novel negative YAP-SAV1 feedback loop in normal lung cells, that was dysregulated in lung cancer cells by smoking-related hypermethylated *SAV1* promoter. Also, we found that the expression of *SAV1* relating to the methylation level of its promoter could be used in diagnosis and therapy of NSCLC.

## Materials and methods

### Chemicals

The antibodies and Chemicals used in this study included Hippo Signaling Antibody Sampler Kit (CST, #8579); GAPDH (CST, #5174); Anti-mouse IgG, HRP-linked Antibody (CST, #7076); Anti-rabbit IgG, HRP-linked Antibody (CST, #7074); YAP (D8H1X) XP® Rabbit mAb (CST, #14074); β-Catenin (D10A8) XP® Rabbit mAb (CST, #8480); Anti-rabbit IgG (H+L), F(ab')2 Fragment (Alexa Fluor® 594 Conjugate) (CST, #8889); Monoclonal Anti-β-Actin antibody (Sigma, A5316); Azacitidine (5-Azacytidine, AZA, 5Aza, S1782) and Verteporfin(S1786) were purchased from Selleck Biochem.

### Cell lines, specimen, and plasmids

All cell lines were from ATCC and cell line authentication was performed by ATCC. HCC827 and H2170 cells were cultured in a humidified incubator under 5% CO2 in RPMI-1640 media (Invitrogen) supplemented with 10% fetal bovine serum. Specimens were kept at 4 °C until they were ready for processing and sliced into 4 µm thick sections for immunohistochemical staining. 8xGTIIC-luciferase was obtained from Stefano Piccolo (Addgene plasmid # 34615) 27, M50 Super 8x TOPFlash and M51 Super 8x FOPFlash (TOPFlash mutant) were provided by Randall Moon (Addgene plasmid # 12456) 28.

### Establishment of stable cell line

Using the lentiviral packaging kit (Genechem Shanghai, China), HEK293T cells were transfected with target plasmids and viral packaging plasmids to generate lentiviral particles. Lentivirus-containing supernatants were harvested 48 h post-transfection. The lentiviruses were then used to infect lung cells which were selected in 2 μg/ml puromyscin selection media.

### Soft agar colony formation assay

For soft agar colony formation assays, 1000 cells were seeded in 6-well plates containing 0.6% bottom layer and 0.3% top layer soft agar. After 10-15 days of growth, colonies (>50 cells) were counted and photographed under the microscope.

### Recombinant plasmids construction

The recombinant luciferase reporter plasmids with different fragments of* SAV1* promoter were constructed using the GV354 vector, a dual-luciferase plasmid containing both firefly luciferase gene and Renilla luciferase gene, was obtained from Shanghai Genechem. First, the different DNA fragments containing the TEAD binding site locating in *SAV1* promoter were cloned using PCR. Then, the target fragment was cloned into GV354 using T4 DNA ligase. The recombinant luciferase reporter plasmids were verified by the DNA sequencing of Huada Genomics Institute (BGI, Guangzhou, China).

### Dual-luciferase reporter assay

A dual-luciferase reporter assay was performed following the manufacturer's instructions using the Promega luciferase reporter system (E1980). To evaluate the WNT pathway, 1×10^4^ 293T cells grown in a 96-well plate were co-transfected with TOPflash (mutant or wildtype, 1 µg) and pRL-TK (50 ng). After 24 hours, the cells were collected using PLB (passive lysis buffer) and a plate-reading luminometer capable of processing a 96-well plate was used to measure luciferase activity as follows: i) Luciferase Assay Reagent II was injected; ii) firefly luciferase activity was measured; iii) Stop & Glo® Reagent was injected and; iv) Renilla luciferase activity was measured. Similarly, using 8xGTIIC-luciferase plasmid (Addgene, #34615) with 8 binding sites of the TEAD, YAP/TAZ-responsive synthetic promoter driving luciferase expression was evaluated based on the aforesaid procedure. All luciferase assays were analyzed after 48h of transfection and normalized to Renilla luciferase.

### RNA extraction and quantitative reverse transcription (qRT)-PCR assays

Extraction of total RNA from tissues and cultured cells was performed using Trizol (Invitrogen) following the manufacturer's protocol. RNA was reverse transcribed using the RevertAid RT Reverse Transcription Kit (Thermo Scientific). Quantitative PCR was performed with PowerUp™ SYBR™ Green Master Mix (Thermo Scientific). The results were normalized with GAPDH. Fold change was determined as 2^-ΔΔCt^ in gene expression.

### ChIP-qPCR

The ChIP assay was performed using the EZ-CHIP^TM^ chromatin immunoprecipitation kit (Merck Millipore, Cat. no. 17-371). In brief: Proteins were cross-linked to DNA by adding formaldehyde directly to the RPMI 1640 culture medium to a final concentration of 1%, and incubated for 10 minutes at room temperature. Then, the cells were washed and collected in ice-cold phosphate-buffered saline (PBS) containing Protease Inhibitor Cocktail II. Cells were pelleted and resuspended in lysis buffer containing Protease Inhibitor Cocktail II. The resulting lysate was sonicated to reduce the size of DNA to approximately 200-1000 base pairs in length. The sample was centrifuged to remove cell debris and diluted ten-fold in ChIP dilution buffer containing Protease Inhibitor Cocktail II. Samples were kept on ice all the time. A 5 μl sample of the supernatant was retained as “Input” and stored at 4 °C. Then 5 µg of antibodies were added to the chromatin solution and incubated overnight at 4 °C with rotation. After antibody incubation, protein G agarose was added and the sample incubated at 4 °C with rotation for an additional 2 h. The protein/DNA complexes were washed with Wash Buffers four times and eluted with ChIP Elution Buffer. Cross-links were then reversed to free DNA by the addition of 5M NaCl and incubation at 65 °C for 4 h. The DNA was purified according to the manufacturer's instructions. 50 μl of DNA was obtained for each treatment. qRT-PCR was performed with 0.2 μl of DNA from each group. The results were calculated by normalizing to the input. Primers for ChIP-qPCR.

For *SAV1* promoter: SiteWt1# Forward: 5'-CCGCCTTCATACCCTGATGT-3', Reverse: 5'-TGGTGCAGATCTCCCACAATTC-3'; Site Wt2#: Forward: 5'-GAGCCTTCTCTGGTCCCTCT-3', Reverse: 5'- GCAAGACTGCCAAATGTACAGG-3'.

### Promoter DNA methylation analysis

Bisulfite genomic sequencing analyses were realized. First, genomic DNA was purified using Tissue DNA Kit (Omega). Using the obtained genomic DNA, the promoter region of *SAV1* gene was amplified by PCR. The PCR primers were in the following:

SAV1-F: 5'-TCTTGCATTCTTCCGTTT-3', SAV1-R: 5'-TCTTATGGGCTTTCAATCAACGA-3'. Sequencing primers were in the following: SAV1-NS-F: 5'-TCTTGCATTCTTCCGTTT-3', SAV1-NS-R: 5'-TCTTATGGGCTTTCAATCAACGA-3'. Then, Conversion of unmethylated cytosines to uracil of *SAV1* promoter region was carried out as described in EZ DNA Methylation-Gold Kit (ZYMO Research). The above products were amplified by PCR using the following primers: SAV1-MNT-F: 5'- TGAGTAGTGTTGTAGA-3', SAV1-MNT-R: 5'- CTAAACTACCTCCCTAAA-3'. At last, the PCR products were sequenced. Bisulfite sequencing primers were in the following: SAV1-MS-F: 5'-TGAGTAGTGTTGTAGAG-3', SAV1-MS-R: 5'-TAAACTACCTCCCTAAAACTCC-3'.

### Sphere formation assays

The sphere formation assay was performed based on the previously described experiment flow elsewhere 29. In brief, lung cancer cell suspension at 1 × 10^3^/well concentration was plated in 24-well ultra-low attachment plate (Corning, 3473). The cells grew in serum-free medium (DMEM/F12) containing 10 μg/L FGF, 20 μg/L EGF, 2% B27 and 5 mg/ml insulin. The formative spheres were counted and the photographs were captured after 2 weeks.

### *In vivo* Subcutaneous Xenograft Model

*In vivo* subcutaneous xenograft model was established to evaluate the tumor growth. Four-week-old female nude mice were randomly assigned to several groups (n=5/group) and subcutaneously injected with 5×10^6^ lung cancer cells in the armpit. After the injection of 1week, every 2 day measured the tumor size using vernier caliper. The tumor volume was estimated using the following formula: V=Width^2^ × Length/2. The tumor weight of each mouse was obtained at the experimental endpoint. All animal experiments were done in accordance with the local animal ethics committee.

### *In vivo* metastasis assays

For metastasis assays, isometric cells of 1×10^6^ were injected into the caudal vein of the mouse by intravenous injection (I.V.). After 8 weeks of monitor, all mice were sacrificed, then the main organs were harvested to assess metastasis. All animal experiments were done in accordance with the local animal ethics committee.

### Organoid establishment and In-vitro drug studies

Based on the guidance of the product manual 30-32, tumor tissues were processed into 2-mm-diameter pieces and washed with culture medium for organoid cultures. Tumor pieces were dissociated into single cells in Advanced DMEMF12 (Gibico) with collagenase (Sigma, C9407) for 1 hour in 37 °C with gentle shaking. Single-cell suspension mixing with Matrigel matrix (Corning, #356231) was seeded into the 24-well ultra-low attachment plate (Corning, 3473). An appropriate proportion of FGF10 (Peprotech, #100-26-100 ug), FGF7 (Miltenyibiotec, #13-097-173-50 ug) and B27 (Invitrogen, #17504-44) were added. Maintain the organoids by refreshing the media every 3 days and passage 1:3 or 1:5 as the lumen becomes filled with dead cells approximately every 7 days. The formed organoids were captured and counted under the optical microscope. For lentiviral infection in lung organoid culture, organoid fragments were prepared with TrypLE Express (Invitrogen, 12605036). Combine organoid fragments with 250 µl of the lentiviral solution and virus was produced using the lentiviral packaging kit (Genechem Shanghai, China). Incubator for 6 hours, seeded the infected organoid fragments and selected in 2 μg/ml puromyscin selection media. For western blot, matrigel/organoid suspension was dissociated with TrypLE Express and organoid pellets were lysed with RIPA buffer (Sigma) with phenylmethylsulfonylfluoride, sodium vanadate, and protease inhibitor cocktail (Roche). Protein was quantified via Bradford assay (Bio-Rad), denatured in sample buffer (Bio-Rad), and loaded for SDS-PAGE. *In vitro* drug studies, organoids were dissociated into single cells, counted, and plated in Matrigel-coated 96 well plates (3,000 cells per well) in triplicate for 24 hours before drug treatment. Organoids were treated with a range of drug concentrations (0.01- 10 mmol/L) for 96 hours and cell viability was determined by CellTiter Glo 3D viability assay (Promega). Drug-response curves were graphed and IC50 values were calculated using Graphpad Prism 7.

### Statistical analysis

Statistical analysis in this study relied on SPSS 23.0 (SPSS, USA) software and GraphPad Prism 7 (GraphPad, USA). All data were presented as mean ± SD. Student's t-test and one-way ANOVA were applied in two or more than two-group comparisons. P < 0.05 was considered statistically. All experiments were duplicated as equal or greater than 3 times.

## Results

### YAP-SAV1 negative feedback loop maintains lung epithelial cell homeostasis

**Figure 1:**
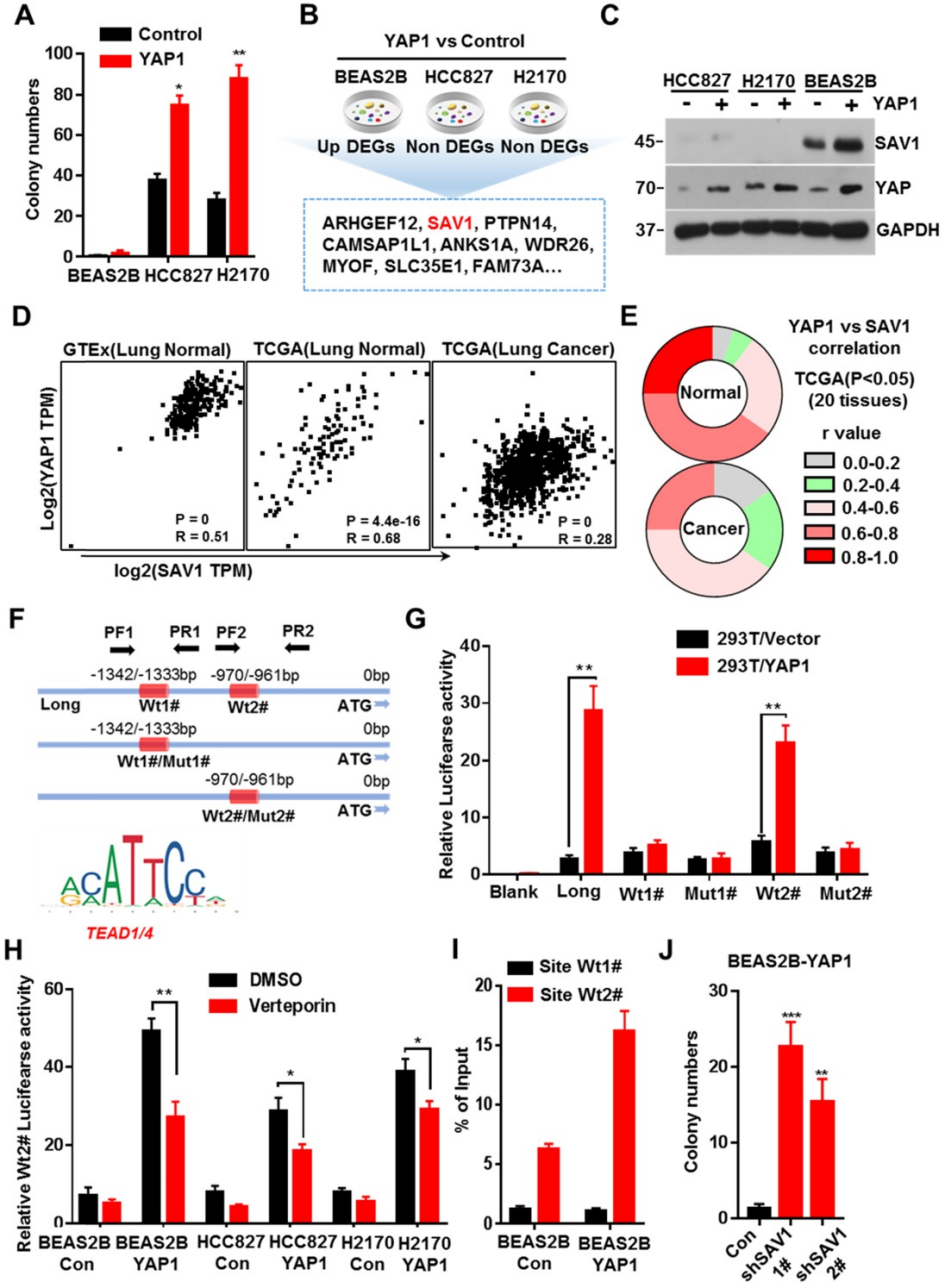
** YAP-SAV1 negative feedback loop maintains lung epithelial cell homeostasis. (A)** Quantification of colony numbers of different lung cell lines in soft agar. **(B)** Intersection of genes up-regulated in BEAS2B cells with those non-significantly upregulated in HCC827/H2170 cells. **(C)** After overexpression of YAP, the expression of the target gene SAV1 was measured by western blot (WB) analysis. **(D)** Using GTEx and TCGA transcriptome data, the transcripts per million (TPM) expression value shows the positive correlation between SAV1 and YAP in normal lung samples rather than in cancer samples. **(E)** TCGA data shows a stronger correlation between SAV1 and YAP in normal tissue than cancer group in 20 tissues. **(F)** Schematic of SAV1 promoter region and the two TEAD binding sites (Wt1#, Wt2#) with two corresponding mutant sites are shown (Mut1#, Mut2#). **(G)** SAV1 promoter activity was assessed with the dual luciferase reporter assay in HEK-293T transfected with indicated plasmids. **(H)** Dual-Luciferase reporter assay in different lung cell lines. Verteporin: YAP-TEAD inhibitor. **(I)** CHIP-qPCR confirmed that Wt2# is the effective binding site located in the SAV1 promoter region rather than Wt1#. **(J)** Quantification of colony numbers of YAP-overexpressing BEAS2B cells with or without SAV1 knockdown in soft agar. Results represent means ± SD of at least two independent experiments. Statistical significance: P< 0.05 (Student's t-test).

Previous studies discovered that exogenous expression of YAP in normal mouse lung cells promotes growth, but fails to induce tumor formation 18. Here, we exogenously expressed YAP in human lung normal epithelial cells BEAS2B *in vitro*, and no malignant transformation was observed, thus inducing no colony formation in soft agar (Fig. 1A, Supplementary Fig. S1A). Considering the important role of the YAP negative feedback mechanism in the maintenance of cell homeostasis, a feedback mechanism regulating YAP activity in normal lung epithelial cells was inferred, which could be dysregulated in lung cancer cells. Therefore, after exogenously expressing *YAP1* gene, we analyzed the differential expressed genes (DEGs) that were up-regulated in normal lung epithelial cells but remained unchanged in lung cancer cells. Through RNA sequencing (RNA-seq) and Venn plotting analysis, 115 significantly altered genes were identified (Fig. 1B). Given the more strongly elevated, SAV1, an upstream regulator of the Hippo signaling pathway that inhibited YAP activity, was first selected for in-depth investigation. The expression of SAV1 was significantly upregulated in BEAS2B cells overexpressing YAP1, however, there was no upregulation in the expression of YAP1 overexpressing lung adenocarcinoma cells HCC827 and lung squamous cell carcinoma cells H2170 (Fig. 1C). Subsequently, we assessed the expression of SAV1 and the common downstream progression-inducing genes of YAP including CTGF and Cyr61 by qPCR. As a result, they were all elevated after overexpressing YAP1 in BEAS2B cells, but SAV1 was not upregulated in HCC827 or H2170 cells (Fig. S1B). Further, we analyzed the correlation between the expression of SAV1 and YAP1 in normal lung tissues in the GTEx database as well as normal lung tissues and lung cancer tissues in the TCGA database. It was found that the expression of SAV1 and YAP1 was highly and positively correlated in normal lung tissues compared to lung cancer tissues (Fig. 1D, Fig. S1E). Further analysis of the correlation between SAV1 and YAP1 expression in 28 different tissues revealed that in 20 different tissues, the expression of SAV1 and YAP1 was significantly and positively correlated in normal tissues, but the positive correlation was not significant in tumor tissues. Besides, the YAP homologue TAZ showed a similar effect (Fig. 1E, Fig. S1C-D). These phenomena prompted this study to evaluate whether YAP is a potential regulator of SAV1. We analyzed the promoter region of SAV1 and found two binding sites (WT1# and WT2#) of TEAD as the major transcription factor (TF) to which YAP targets. The luciferase reporter assays revealed that YAP regulated SAV1 activity while WT2# was the major regulatory region. The use of verteporin, YAP-TEAD binding inhibitor, confirmed that YAP affects SAV1 according to TEAD (Fig. 1F-I). Lastly, we knocked down SAV1 by shRNA in the YAP-overexpress BEAS2B cell line and found that it rescued the colony formation in soft agar (Fig. 1J, Fig. SF-G). As documented, SAV1, an upstream effector of Hippo signaling, is a suppressor of YAP. In this paper, we found that YAP reversely regulated SAV1. Further, we demonstrated that YAP-activation directly induced the expression of its negative regulator SAV1 complexed with the transcription factor TEAD (TEA domain family member), to constitute a negative feedback loop of the Hippo pathway and maintain lung epithelial cell homeostasis, but somehow it was dysregulated in lung cancer cells.

### SAV1 promoter methylation inhibits YAP-SAV1 negative feedback regulation in lung cancer cells

**Figure 2:**
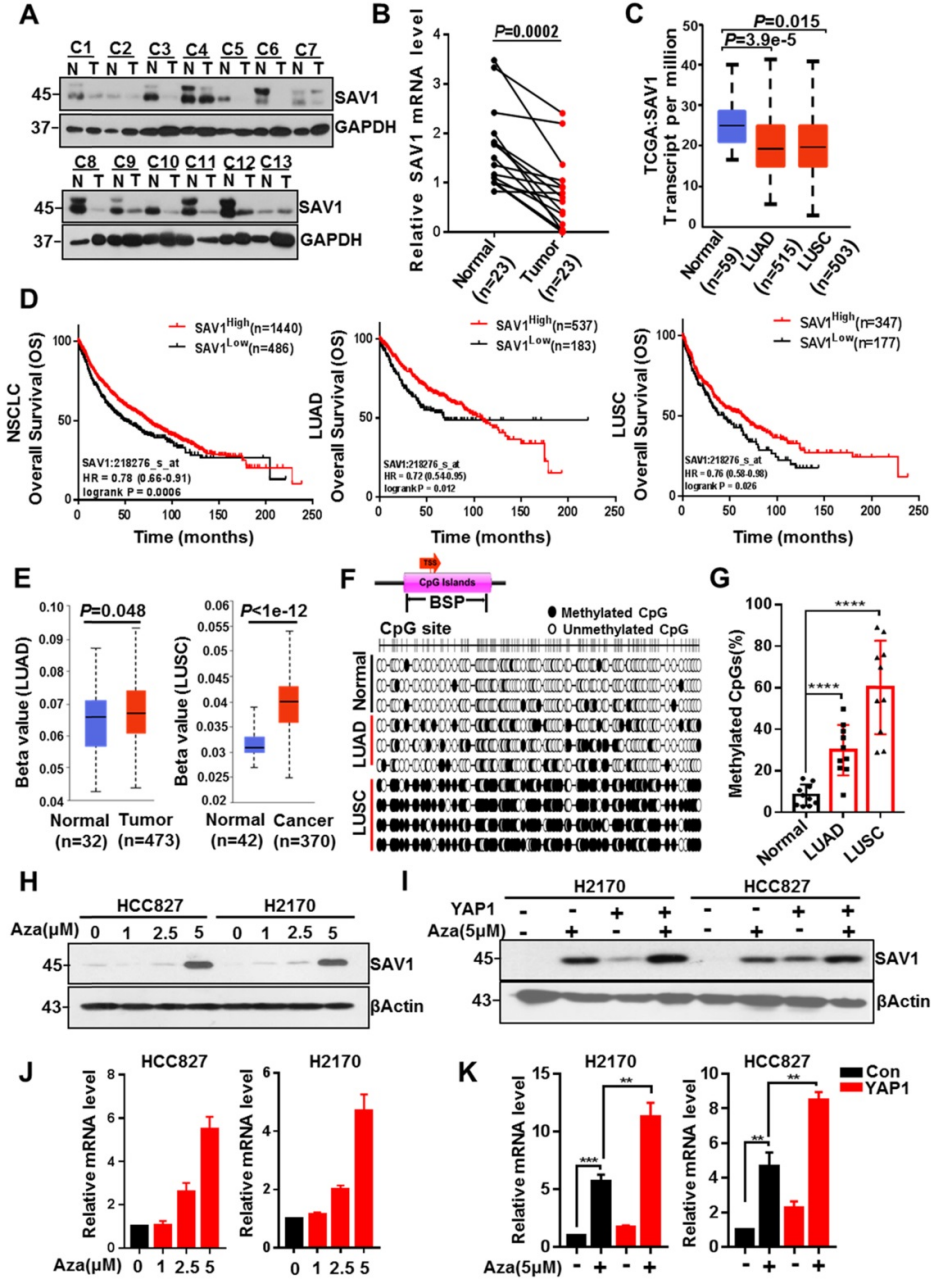
** SAV1 promoter methylation inhibits YAP-SAV1 negative feedback regulation in lung cancer cells. (A)** Western blot (WB) analysis of SAV1 protein expression in lung cancer and adjacent healthy tissue samples.** (B)** The relative mRNA expression level of SAV1 in lung cancer and adjacent healthy tissue samples was analyzed by QPT-PCR.** (C)** The relative mRNA expression level of SAV1 in normal tissues (n=59), LUAD (n=515) and LUSC (n=503) was analyzed by cancer genome Atlas (TCGA). **(D)** Kaplan-meier overall survival curves of lung cancer patients with low and high SAV1 expression were obtained from TCGA database. (E) Using the Human lung Methylation450K BeadChips data to assess SAV1 promotor methylation level.** (F, G)** The DNA methylation at the SAV1 promoter (indicated region) measured by bisulfite sequencing **(H, J)** Treated HCC827 and H2170 cells with various concentrations of AZA, then SAV1 protein and mRNA levels were determined by WB and qRT-PCR, respectively. **(I, K)** The protein and mRNA expression levels of SAV1 in the presence and absence of YAP and AZA were detected by WB and qRT-PCR, respectively. Results represent means ± SD of at least two independent experiments. Statistical significance: p< 0.05 (Student's t-test).

To explore why the YAP-SAV1 negative feedback loop was dysregulated in lung cancer cells, firstly, we used clinical lung samples and confirmed lower expression of SAV1 in cancer tissue versus normal tissue (Fig. 2A-B, Fig. S2A). Relative TCGA data and lung cell lines demonstrated a similar tendency (Fig. 2C, Fig. S2B). Furthermore, high SAV1 expression conferred better overall survival (OS) in non-small cell lung cancer (NSCLC) comprising lung adenocarcinoma (LUAD) and lung squamous carcinoma (LUSC) (Fig. 2D). DNA methylation is a vital epigenetic modification regulating gene expression in development and disease, besides, its changes are highly associated with lung cancer 33. Thus, the present study evaluated whether the altered SAV1 expression levels were associated with the methylation status of the promoter. Using the Human lung Methylation450K BeadChips data to assess SAV1 promotor methylation level 34, the SAV1 promoter was hypermethylated in lung cancer tissues compared to normal tissues (Fig. 2E). Additionally, analysis of the genome-wide DNA methylation data of LUAD and LUSC samples obtained from the TCGA dataset (http://cancergenome.nih.gov/) found 14 cg sites on the SAV1 gene. In LUSC, 10 sites were hypermethylated in tumor tissues compared to normal tissues, while only 3 sites in LUAD had methylation differences (Fig. 2D). Further, bisulfite sequencing PCR (BSP) was used to analyze normal lung and lung cancer tissues where additional methylated CpG islands of SAV1 promoter were determined in cancer. As shown, this phenomenon was particularly evident in LUSC (Fig. 2G, Fig. S2C-D). To validate whether methylation affected the expression of SAV1, HCC827 and H2170 were treated with 5 μM of AZA, a methylation inhibitor for 72h, then we detected the expression of SAV1 in protein and mRNA level. Results revealed that inhibition of methylation in the promoter region of SAV1 promoted the transcriptional activation of SAV1 (Fig. 2H-I). Reports indicate that, as a transcriptional cofactor, YAP is frequently combined with transcription factors in the gene promoter region or remotely regulates the expression of the target gene. Therefore, we evaluated whether SAV1 promoter methylation influenced transcriptional regulation of YAP in lung cancer cells. As a result, during AZA treatment, exogenous expression of YAP significantly increased SAV1 transcription and protein expression (Fig. 2J-K). This suggested that the methylation of the SAV1 promoter region in lung cancer cells inhibited transcriptional regulation of YAP.

### Smoking triggers the methylation of SAV1 and MB1/2 could be used in lung cancer diagnosis

**Figure 3:**
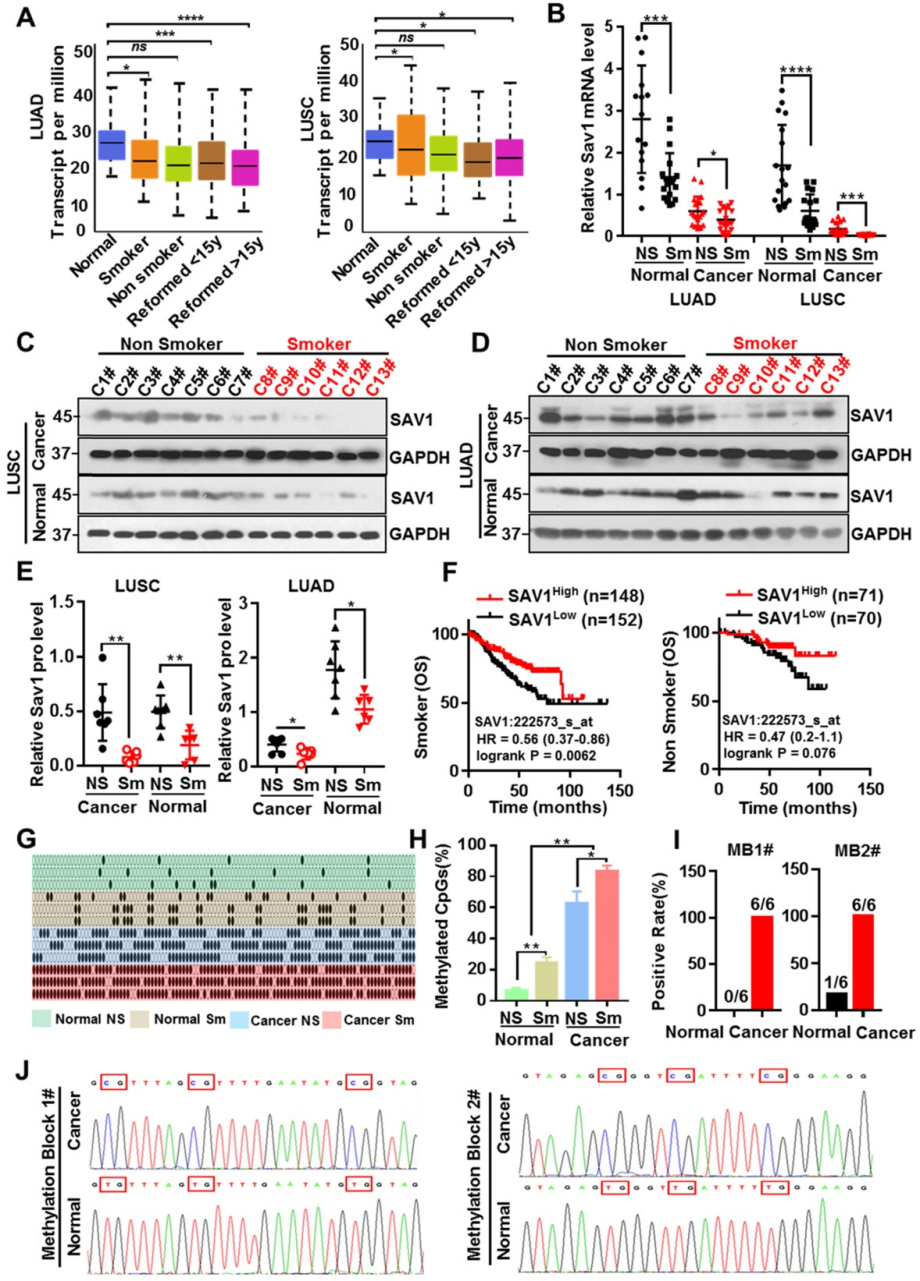
** Smoking triggers the methylation of SAV1 and MB1/2 could be used in lung cancer diagnosis. (A)** SAV1 gene expression data (normalized TPM) for smoking and non-smoking patients were obtained from the TCGA LUAD/LUSC samples. **(B)** The relative SAV1 mRNA expression level was determined by RT-qPCR in lung cancer tissue and adjacent healthy tissue samples from smoking or non-smoking patients with LUAD/LUSC. **(C, D)** Western blot (WB) showing expression levels of SAV1 in lung cancer tissue and adjacent healthy tissue samples from smoking or non-smoking patients with LUAD/LUSC. **(E)** Quantification of WB band intensity from (C,D). **(F)** Kaplan-Meier patient survival curves for overall survival (OS) rates based on SAV1 expression in smoking or non-smoking patients from TCGA database. **(G, H)** The DNA methylation level of SAV1 promoter region was analyzed by Bisulfite sequencing PCR (BSP) in lung cancer tissue and adjacent healthy tissue samples from smoking or non-smoking patients. **(I, J)** Two methylation blocks (MB1, MB2), CpG clustering sites locating at SAV1 promoter specifically related to lung cancer, were found by BSP. Results represent means ± SD of at least two independent experiments. Statistical significance: p< 0.05 (Student's t-test).

Tobacco smoking is the largest preventable cause of lung cancer and smoking has been the most informative environmental correlate of DNA methylation, which in turn affects the occurrence and progression of tumors. To evaluate whether smoking causes methylation of the SAV1 promoter region affecting its expression, the correlation between smoking and SAV1 expression was initially analyzed. Analysis of the LUAD and LUSC samples obtained from the TCGA dataset (http://cancergenome.nih.gov/) found that SAV1 was at lower expression in smokers and this was more predominant in LUSC-smokers (Fig. 3A), we also used clinical lung samples and confirmed a similar tendency in both protein and mRNA levels (Fig. 3B-E). We found smoking cancer patients with high expression of SAV1 had a higher OS rate and exhibited a slight trend in the nonsmokers (Fig. 3F), implying that smoking cancer patients with higher SAV1 appeared to have a better prognosis. For this close relationship between smoking and SAV1, the relationship between smoking and methylation of SAV1 was explored. Bisulfite sequencing PCR (BSP) was used to detect the methylation changes in the SAV1 promoter region in smoking lung cancer and non-smoking lung cancer tissues, where much more methylated CpG sites of SAV1 promoter were discovered in smoking cancer patients. Further through the combinatorial analysis of the differences between adjacent CpG clustering sites in normal and lung cancer tissues, two clustering sites (MB1 and MB2, Methylated Blocks) were discovered, specifically related to lung cancer tissues. Analysis of clinical case combing with MB1 and MB2 demonstrated that they can be used to distinguish between tissue types and smoking habits (Fig. 3G-J, Table 1). Since DNA methylation is considered a potential biomarker for cancer prognosis and diagnosis, MB1 and MB2 can be used in the diagnosis of smoking-associated lung cancer.

### Exogenous SAV1 inhibits the malignancy of Lung Cancer Cell

**Figure 4:**
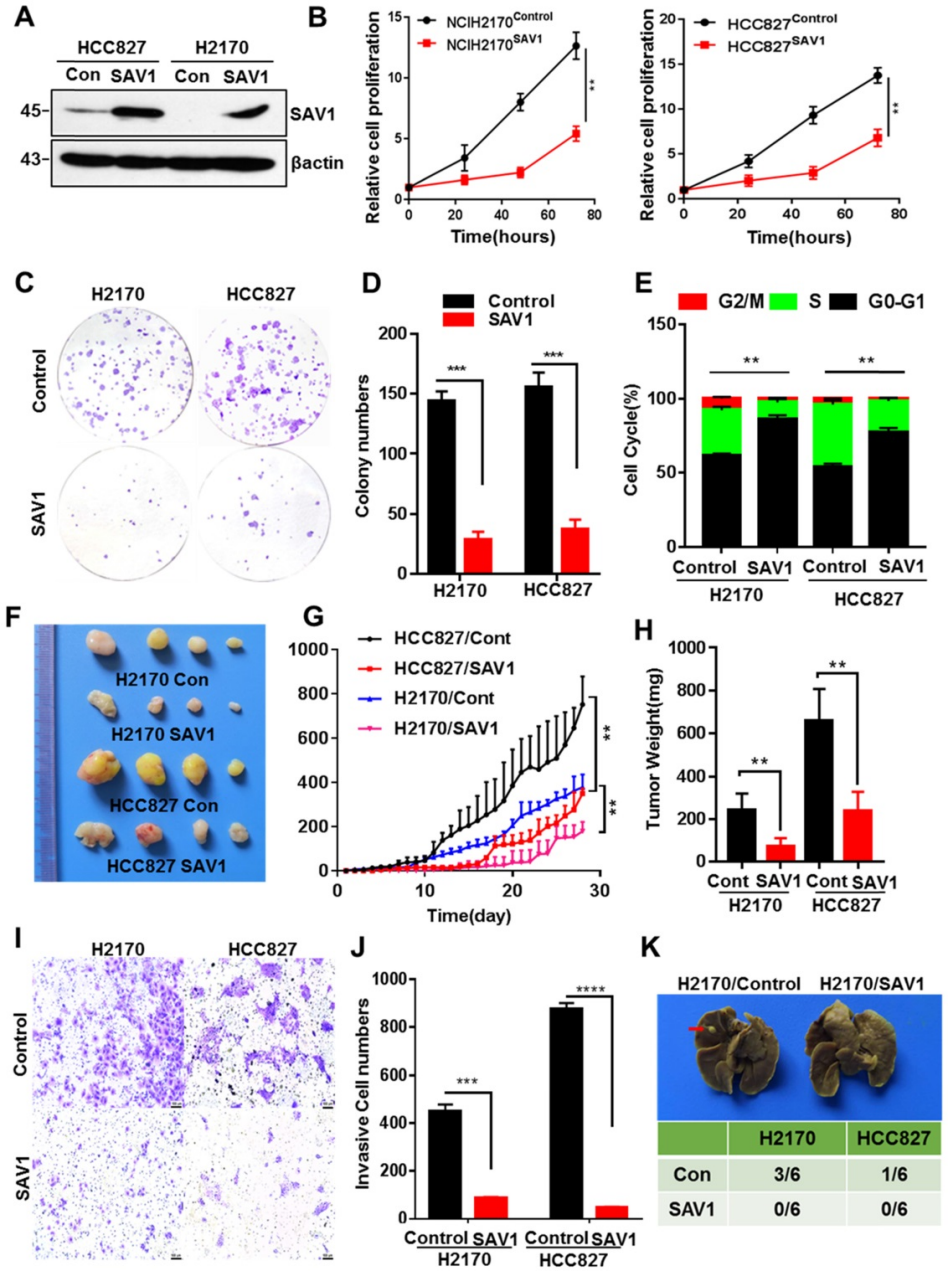
** Exogenous expression of *SAV1* inhibits the malignancy of Lung Cancer Cell. (A)** The verification of exogenously expressed SAV1 cell line by WB. **(B)** Viable cell counts were performed, and proliferation was determined by living cells workstation. **(C, D)** Plate clone formation assay was performed to detect the cell proliferative ability. **(E)** Using propidium iodide (PI) staining, cell cycle analysis was conducted by flow cytometry and the results were analyzed by flowjo software. (F) Images of subcutaneous xenograft tumors formed by lung cancer cells with expression change of SAV1. **(G, H)** Tumor volume changes were monitored and end-point tumor weight was measured as shown. **(I, J)** Representative images of the Transwell assay (magnification, ×200) and quantification of the Transwell assay. Scale bar: 100 µm. **(K)** Lung cancer cells were intravenously injected into nude mice via the lateral tail vein and the metastasized foci at the lung surface are shown. Results represent means ± SD of at least two independent experiments. Statistical significance: p< 0.05 (Student's t-test).

To further analyze the function of SAV1 in lung cancer, we exogenously expressed SAV1 in lung cancer cell lines and found that exogenous SAV1 repressed the proliferative ability of lung cancer cells *in vitro* (Fig. 4A-E, Fig. S4A). Using immunodeficient nude mice, the tumorigenic role of SAV1 in lung cancer cells was assessed *in vivo*. As a consequence, the xenograft tumor formation ability was decreased in the SAV1-overexpressed group (Fig. 4F-H). Further, we sought to understand the cellular function of SAV1 in tumor invasion and metastasis. Transwell assay and scratch wound assay revealed that exogenous SAV1 in lung cancer cells restrained the invasive ability and migration. Moreover, after inoculating lung cancer cells via caudal vein, the metastatic rate of lung in mice of control was higher compared to the experimental groups (Fig. 4I-K, Fig. S4B-C). As such, we acknowledged that the malignancy of lung cancer can be reduced by overexpressing SAV1.

### Exogenous expression of SAV1 inhibits YAP transcriptional activity by stabilizing MST1 protein

**Figure 5:**
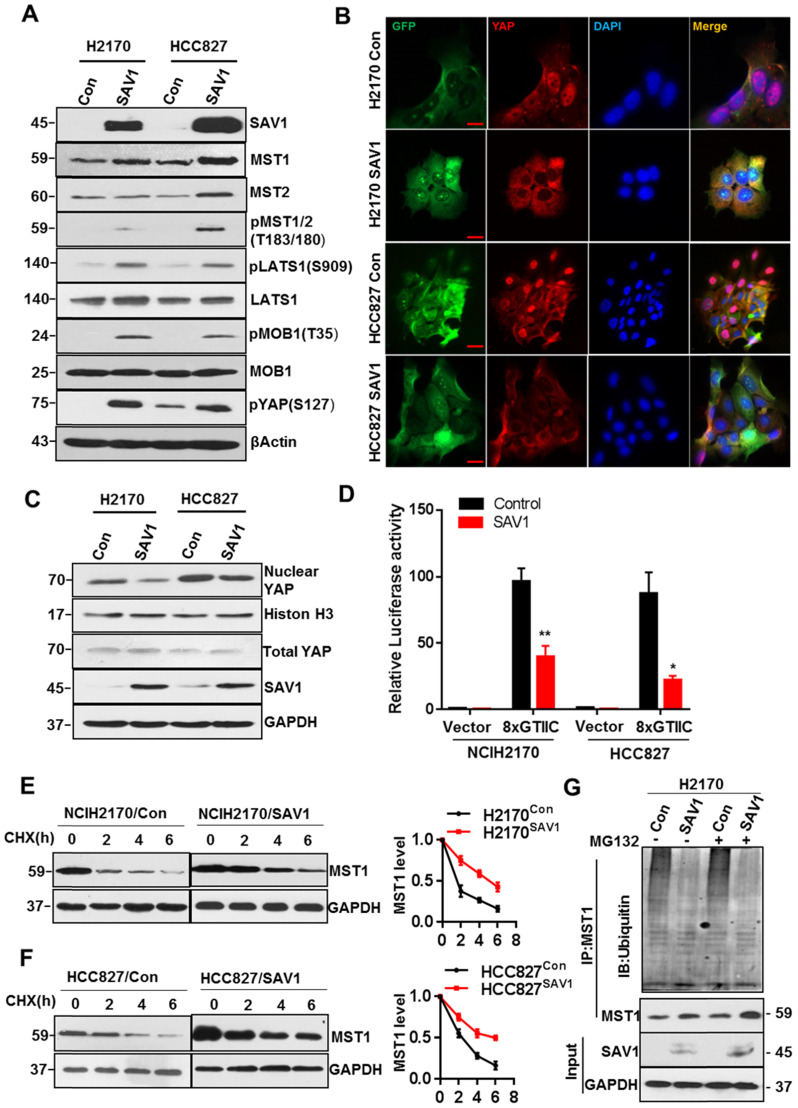
** Exogenous expression of *SAV1* inhibits YAP transcriptional activity by stabilizing MST1 protein. (A)** WB was used to analyze the effect of SAV1 overexpression on Hippo signaling pathway components in H2170 and HCC827 cells. **(B)** The effect of SAV1 overexpression on the subcellular localization of YAP was determined by immunofluorescence (IF) staining for endogenous YAP (red) along with DAPI for DNA (blue), Scale bar: 50 µm. **(C)** The effect of SAV1 overexpression on the subcellular localization of YAP was determined by WB. **(D)** Luciferase reporter assay (8XGTIIC) in NSCLC cell lines transfected with SAV1 and without. **(E, F)** Lung cancer cell lines with overexpression and none-overexpression SAV1 were treated with cycloheximide (CHX, 200 µM) for 0, 2, 4, and 6 hours, and lysates were subjected to western blot as indicated. GAPDH was used as the loading control. (Right) The relative quantitation of MST1 after CHX treatment. **(G)** Immunoprecipitation of MST1 protein in overexpression and none-overexpression SAV1 cell lines and determination of its ubiquitination by immunoblotting with anti-ubiquitin in the presence or absence of the proteasome inhibitor MG132 (50 µM). Results represent means ± SD of at least two independent experiments. Statistical significance: p< 0.05 (Student's t-test).

To elucidate the mechanism of exogenous SAV1 in inhibiting the malignancy of lung cancer cells and considering that SAV1 is a known suppressor of YAP, we first investigated whether SAV1 affects the components of the Hippo pathway. Based on WB results, the key components including pMST1/2, pLATS1, pMOB1, pYAP of the Hippo pathway were elevated by exogenous SAV1 (Fig. 5A). Then, we found YAP nuclear exclusion and cytoplasmic retention in SAV1 overexpressing lung cancer cell line compared to the control group (Fig. 5B-C). Further, YAP activities in lung cancer cells were restrained by SAV1 taking use of YAP/TAZ-responsive TEAD reporter (8xGTIIC-luciferase reporter) and qPCR to measure the expression of YAP target genes (Fig. 5D, Supplementary Fig. S5A). It has been reported that SAV1 interacts with MST1, a major downstream component of SAV1 in the Hippo pathway which protects SAV1 from degradations 35. Also, the WB results ahead revealed that MST1 was upregulated by SAV1 overexpression, prompting this work to explore whether SAV1 exerts a similar role to MST1. Cycloheximide (CHX) was added to the culture medium to inhibit protein synthesis and the turnover of MST1 was analyzed by immunoblotting of the cell lysates collected at 0, 2, and 6 h. It appeared that SAV1 stabilized MST1 in both lung cancer cell lines, H2170 and HCC827 (Fig. 5E-F). Immunoprecipitation (IP) results suggested the deubiquitination/stabilization of MST1 by SAV1 (Fig. 5G). The above results demonstrated that SAV1 interacted with and stabilized MST1, activating the canonical Hippo signaling pathway, hence the nuclear translocation of YAP was restrained, thereby reducing transcriptional activity of YAP.

### Exogenous expression of SAV1 attenuate lung cancer stem cell traits by inhibiting WNT signaling

**Figure 6:**
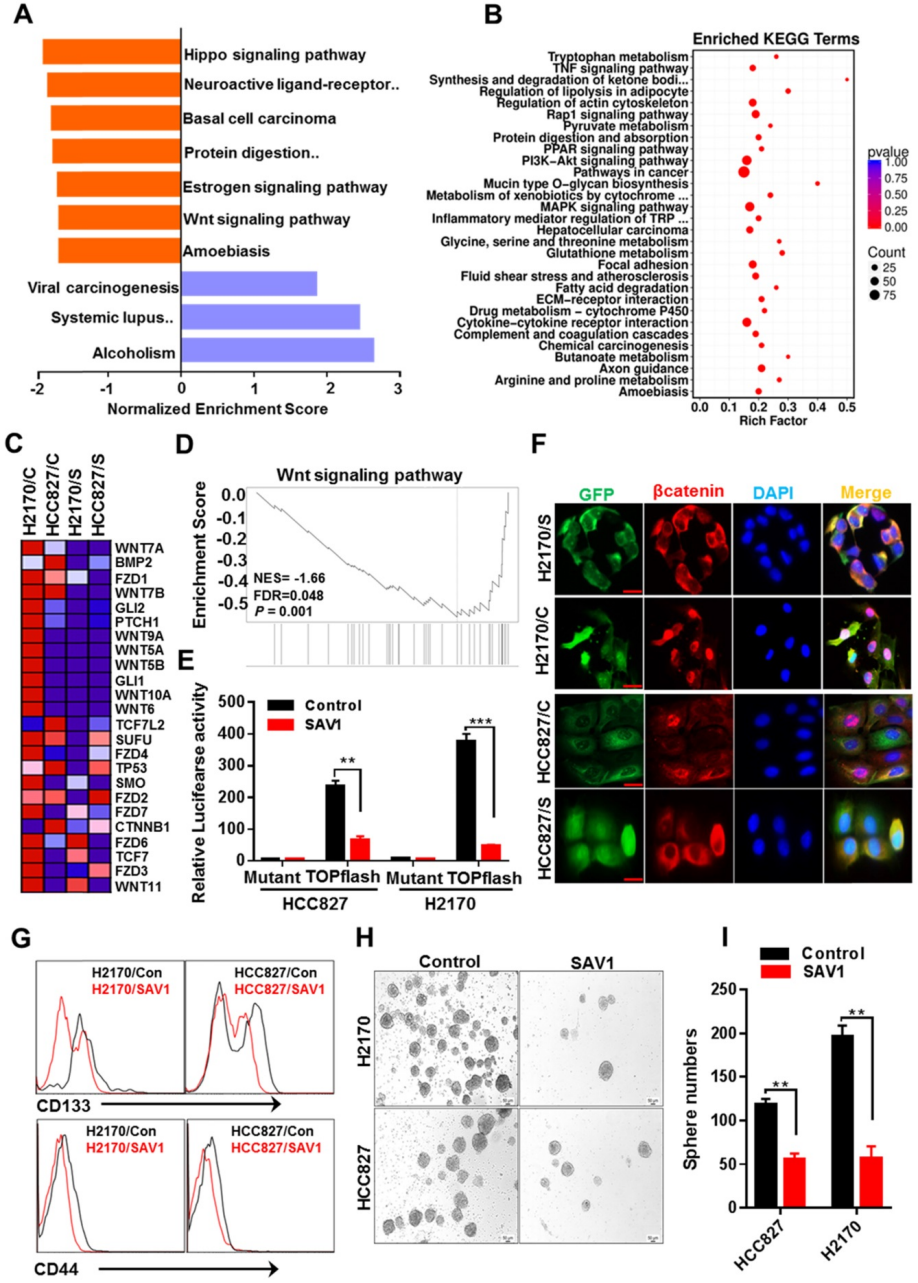
** Exogenous expression of *SAV1* attenuates lung cancer stem cell traits by inhibiting WNT signaling. (A)** Gene set enrichment analysis (GSEA) was performed on the expressed genes found in NSCLC cell lines with and without forced SAV1 expression, and the top 10 GSEA gene clusters were displayed. **(B)** KEGG pathway enrichment analysis of differentially expressed transcripts. **(C, D)** Heatmap (C) shows the differentially expressed genes related to the WNT signaling pathway in NSCLC cell lines, and the enrichment score (D) was shown. C represents control; S represents transduced SAV1. **(E)** Luciferase reporter assay (TOPflash) in NSCLC cell lines transfected with SAV1 and without. **(F)** The effect of SAV1 on the subcellular localization of β-catenin was determined by immunofluorescence (IF) staining for endogenous β-catenin (red) along with DAPI for DNA (blue). Scale bar: 50 µm. **(G)** Flow cytometry analysis of the indicated surface markers on NSCLC cell lines with and without forced SAV1 expression. **(H, I)** Sphere formation assay was performed and the sphere number was measured. Results represent means ± SD of at least two independent experiments. Statistical significance: p< 0.05 (Student's t-test). Scale bar: 50 µm.

Functional gene enrichment analysis of RNA transcriptome data of lung cancer cells reflected that except for activating the Hippo signaling pathway, SAV1 exogenous overexpression also influenced the WNT signaling pathway and downregulated the major components of WNT signaling (Fig. 6A-D). To validate the results above, the TOPflash luciferase reporter assay was performed, which revealed a decreased WNT signaling pathway activity in the stably overexpressed SAV1 cell lines (Fig. 6E). Immunofluorescence outcomes confirmed the decreased levels of Wnt-induced β-catenin nuclear accumulation (Fig. 6F). The WNT signaling pathway has been reported to maintain lung cancer stem cell features. Thus, the expression putative surface markers (CD44, CD133) of stem cells were analyzed in lung cancer cell lines using flow cytometry, where we found that the expression of them was reduced in the overexpressed SAV1 cells compared to the control (Fig. 6G). Additionally, a sphere formation assay was conducted and the results showed the reduced sphere formation ability in the overexpressed-SAV1 group (Fig. 6H-I). Therefore, SAV1 reduced stem cell-like characteristics of lung cancer cells by suppressing nuclear β-catenin accumulation thus inhibiting WNT signaling pathway.

### SAV1 as a potential gene therapy target for smoking-related lung cancer

**Figure 7:**
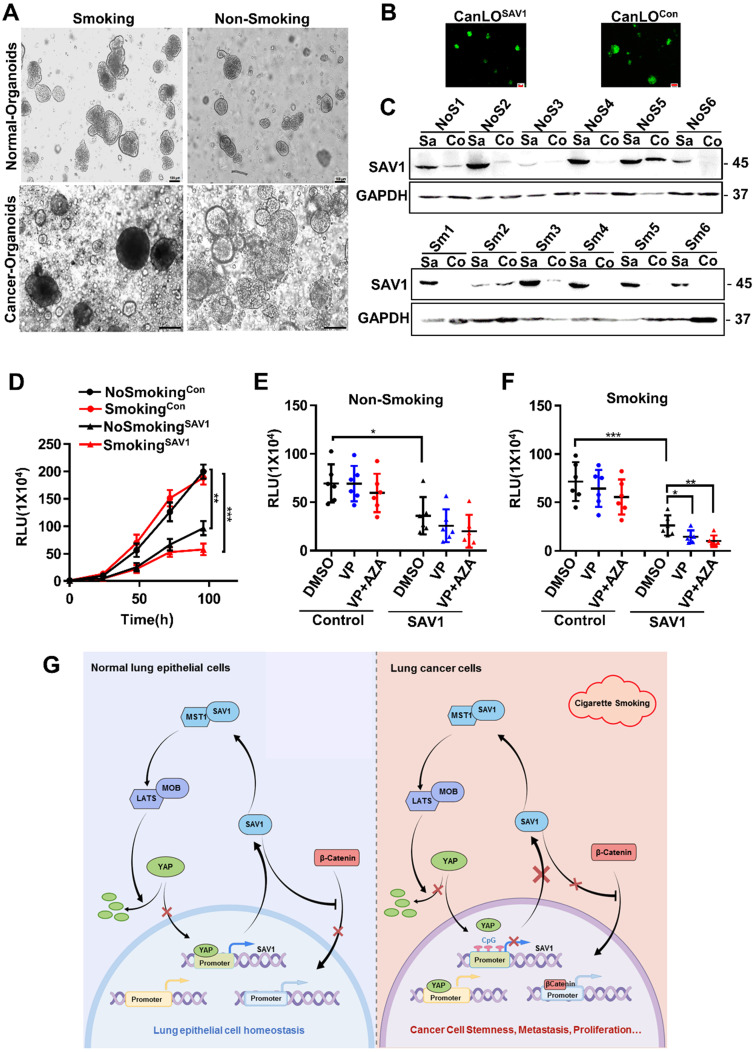
***SAV1* as a potential gene therapy target for smoking-related lung cancer. (A)** The representative picture of lung organoids of lung cancer tissue and adjacent healthy tissue samples from smoking or non-smoking patients. Scale bar: 100 µm. **(B, C)** Exogenously expressed SAV1 was expressed in lung organoids. (B) Fluorescence pictures of the representative organoids. (C) Validation of the overexpression of exogenous SAV1 in lung organoids as assessed via Western blot. Scale bar: 100 µm. Co represents non-transduced SAV1; Sa represents transduced SAV1. NoS represents non-smoking group; Sm represents smoking group. **(D)** Cell Viability Assay, Viability of 3D spheroids (lung organoids) of different groups was evaluated using the CellTiter-Glo® 3D Cell Viability Assay (Promega). **(E, F)** Effects of VP and AZA on lung organoids of different groups. **(G)** Schematic diagram about the mechanism of SAV1 in normal lung epithelial cell and lung cancer cell (Created with figdraw.com). Results represent means ± SD of at least two independent experiments. Statistical significance: p< 0.05 (Student's t-test).

Considering the above-mentioned functions and effects of SAV1, to determine the feasibility of SAV1 as a gene therapy target for lung cancer, a lentivirus-mediated SAV1 gene transfer lung organoid model was established using clinical tissue samples. Fluorescent pictures were obtained and WB assay was performed to validate the effect of SAV1 overexpression (Fig. 7A-C). Using this model, we found that SAV1 overexpression inhibited tumor growth compared to the control and was more profound in the smoking group (Fig. 7D). Further, using AZA, a methylation inhibitor alone regulated tumor growth, whereas, plus Veterporin (VP), a YAP-TEAD inhibitor, the combination of two drugs appeared more effective. The effect was more pronounced in the smoking group (Fig. 7E-F). In summary, exogenously overexpression of SAV1 combined with methylation inhibitor and YAP-TEAD inhibitor is the most effective approach for the smoking NSCLC population.

## Discussion

Negative feedback regulation is a prevalent mechanism for the body to maintain robustness 36. In this study, we identified YAP-SAV1 as a novel negative feedback mechanism: In the Hippo pathway, the nucleus YAP was bound to SAV1 promoter and transcriptionally activated SAV1, SAV1 complexed with MST1 and stabilized it. Through a MSTs/LATSs-kinase signaling cascade, the downstream effector phosphorylated YAP was excluded from the nucleus. Whereas in cancer cells, when smoking or other potential unknown factors prompted the hypermethylation of SAV1 promoter, the nucleus YAP could not incite the expression of SAV1 above mentioned. Therefore, SAV1 was at low expression in cancer cells, compared to normal lung epithelial cells. This finding enriched the Hippo signaling pathway and filled gaps in the pool, hence, a deeper understanding of the Hippo/YAP pathway has been provided (Fig. 7G). Also, it was likely that the YAP1 paralogue TAZ could be playing a similar role. However, a further in-depth research is essential.

Furthermore, we found the hypermethylation of SAV1 promoter in NSCLC, of which the two smoking-associated methylated blocks (MB1 and MB2) were identified, and was more predominant in the smoking-LUSC patient. DNA methylation, a biomarker for lung cancer, is a key event occurring at the early stage of tumors, while hypermethylation of CpG islands is a signature of malignant progression 37. This indicates that the methylation of SAV1 might be utilized in early screening or as a diagnostic marker of LUSC at an early stage.

We found that overexpression of SAV1 in lung cancer cells activated the Hippo signaling, thus YAP was excluded from the nucleus by phosphorylation. Further, SAV1 was bound to and stabilized MST1 by reducing its ubiquitination and degradation. Ubiquitination, an important post-translational modification, is mediated by E1 (ubiquitin-activating), E2 (ubiquitin-conjugating), and E3 (ubiquitin ligase) enzymes. The activity of the ubiquitination system depended on the specificity of E3 ubiquitin ligase. Given that SAV1 reduces ubiquitination and degradation of MST1, we speculated that SAV1 might exert an effect on E3 ubiquitin ligase. Furthermore, we discovered that overexpression of SAV1 downregulated the WNT/β-catenin signaling pathway. The canonical Wnt/β-catenin signaling majorly regulated the development and tissue homeostasis, which is also a key driver of most types of tissue stem cells in adult mammals 38. Therefore, aberrant regulation of this pathway is associated with various diseases, including cancer. Notably, β-catenin is a key regulatory factor for this pathway. Overexpression of SAV1 inhibited β-catenin nuclear translocation, suppressing the WNT/β-catenin pathway. Therefore, the reduced stemness capability was shown. Reports suggest that WNT/β-catenin signaling interacts with Hippo signaling 39, 40. Our findings showed a crosstalk of them and suggested that SAV1 is the traffic protein that connects these two pathways. Additional studies are necessary to exhaustively investigate this crosstalk.

Smoking is one of the most serious social public health problems of global concern. People who have ever smoked are more likely to develop lung cancer and some other smoking-related diseases, even after quitting for many years 10, 41. EGFR-TKIs treatment of NSCLC with EGFR mutations shows high response rates and long PFS. However, smoking status affects the efficiency of EGFR-TKIs in NSCLC patients. Multiple studies have shown that current smokers have lower objective response rates (ORR), PFS, and overall survival (OS) than former smokers and never smokers 42. Mechanically, lung cancer cells taken from the lungs of smokers contain “drive” EGFR mutations and many other “passenger” mutations. These passenger genes may modify signal transduction pathways, making it more difficult to induce cell death with EGFR-TKIs therapy alone 43, 44. In our study, it was found that the expression of SAV1 in lung cancer tissue of smoking patients was significantly lower than that of non-smoking patients, and the lentivirus-mediated SAV1 gene transfer could inhibit the growth of NSCLC cells and organoids, especially in smoking patients. These results suggest that SAV1 gene can be used as a potential gene therapy target for smoking lung cancer patients. In addition, targeted SAV1 gene therapy combined with TKIs therapies to benefit more smokers with lung cancer is a topic worthy of further study.

The treatment of NSCLC has undergone remarkable changes. The therapeutic potential of epigenetics targeted drugs in combination with chemotherapy, targeted therapy and/or immunotherapy is currently being intensively investigated. Using the preclinical lung organoid models, we confirmed that overexpression of SAV1 inhibits solid tumor growth. Under the condition of exogenous overexpression of SAV1, YAP-TEAD inhibitor (VP, Verteporfin) and hypomethylating agents (AZA, Azacytidine) were effective in constraining the tumor growth and the combination of the two drugs was significantly effective. The above-mentioned outcome was predominant in the smoking group. Given that AZA and VP are FDA-approved drugs, this provides a potential and feasible clinical medication regimen for lung cancer patients with low expression of SAV1, particularly the smoking population. In addition, there is accumulating evidence that YAP expression may confer resistance to immunotherapy by upregulating PDL1 expression and by regulating MDSC cells (myeloid-derived suppressor cells) 45, 46. Therefore, it was thought that targeted SAV1 gene therapy combined with immunotherapy could be a valuable tool to increase the survival of NSCLC. Taken together, this SAV1-mediated feedback loop provides an effective mechanism to establish the robustness and homeostasis of YAP regulation, and it can be used as a potential therapeutic target for smokers with NSCLC.

## Supplementary Material

Supplementary methods and figures.

## Figures and Tables

**Table 1:** Clinical performance of the SAV1 DNA methylation biomarker

	n = 30	MB1	P value	MB2	P value
**Age(years)**			0.12		0.69
≥45	21	16/21(76%)		10/21(48%)	
<45	9	4/9(44%)		3/9(33%)	
**Gender**			0.45		0.71
Male	19	12/19(63%)		9/19(47%)	
Female	11	5/11(45%)		4/11(36%)	
**Tissue type**			<0.0001		0.001
Normal (>2 cm)*	30	0/30(0%)		1/30(37%)	
Cancer	30	17/30(57%)		12/30(40%)	
**Smoking habits**			0.0002		0.004
Smoker (Current&Former)	22	17/22(77%)		13/22(59%)	
Non-smoker	8	0/8(0%)		0/8(0%)	
**Histology subtype**			0.14		0.28
Squamous	17	12/17(71%)		9/17(53%)	
Adenocarcinoma	13	5/13(38%)		4/13(31%)	
**Clinical Stage**			0.71		0.71
I - II	12	6/12(50%)		6/12(50%)	
III - IV	18	11/18(61%)		7/18(39%)	

*2 cm from the cancer tissue; Chi-Square (and Fisher's exact) test.
