# Supplementary material for: Smoke-induced SAV1 Gene Promoter Hypermethylation Disrupts YAP Negative Feedback and Promotes Malignant Progression of Non-small Cell Lung Cancer
Source: Int J Biol Sci. 2022 Jul 11;18(11):4497–512. doi: 10.7150/ijbs.73428 (PMC9295071; doi:10.7150/ijbs.73428)
Supplement: Supplementary file 1 — Supplementary methods and figures. [file ijbsv18p4497s1.pdf]

Supplemental Information

Smoke-induced SAV1 gene promoter hypermethylation disrupts YAP negative feedback and promotes malignant progression of non-small cell lung cancer

Supplemental Experimental Procedures

RNA extraction and quantitative real-time PCR analyses (qRT-PCR)

The TRIzol reagent was used to extract total RNA. cDNA was generated with the PrimeScriptRT reagent kit. GAPDH was performed as an internal reference for cytoplasmic gene expression. The expression change of genes was calculated by the $2^{-\Delta\Delta Ct}$ method.

Western Blot

Total protein was extracted from cells using RIPA buffer (Thermo Scientific, Rockford, IL, USA) in the presence of protease inhibitors (Protease Inhibitor Cocktail, Thermo Scientific). The protein concentration of lysates was measured using a BCA Protein Assay Kit (Thermo Scientific). Equivalent amounts of protein were mixed with 5×Lane Marker Reducing Sample Buffer (Thermo Scientific), and resolved by electrophoresis in a 10% SDS–polyacrylamide gel and then transferred onto Immobilon-P Transfer Membrane (Merck Millipore). The membranes were blocked with 5% non-fat milk in Tris-buffered saline and then incubated with the primary antibodies ollowed by secondary antibody. The signal was detected using enhanced chemiluminescence western blot detection kit. The primary antibody for GAPDH (abs132004) was from absin. The primary antibody for SAV1 (#13301) was from Cell Signaling Technology. The primary antibody for β-Actin (A5316) was from sigma.

Flow Cytometry Analysis

Cell cycle experiment was carried out using the cell cycle staining kit (MultiSciences (Lianke) Biotechnology) following the manufacture's instruction. Briefly, cells were trypsinized and incubated with the primary antibody from the kit. The cell cycle was evaluated using flow cytometry.

Immunohistochemistry

The sections were dried at 60°C for 2 h and then deparaffinized in xylene and rehydrated using a series of graded alcohol washes. The tissue slides were then treated with 3% hydrogen peroxide in methanol for 15 min to quench endogenous peroxidase activity and antigen retrieval then performed by incubation in 0.01 M sodium cirate buffer (pH 6.0) and heating using a microwave oven. After a 1 h preincubation in 10% goat serum, the specimens were incubated with primary antibody overnight at 4°C. The tissue slides were treated with a non-biotin horseradish peroxidase detection system according to the manufacturer’s instruction (DAKO, Glostrup, Denmark). Two different pathologists evaluated the immunohistological samples.

Primers

Primers for qRT-PCR

GAPDH

Forward: 5'-ATTCCATGGCACCGTCAAGGCTGA-3'

Reverse: 5'-TTCTCCATGGTGGTGAAGACGCCA-3'

YAP

Forward: 5'-TAGCCCTGCGTAGCCAGTTA-3'

Reverse: 5'-TCATGCTTAGTCCACTGTCTGT-3'

CTGF

Forward: 5'-CAGCATGGACGTTCGTCTG-3'

Reverse: 5'-AACCACGGTTTGGTCCTTGG-3'

CYR61

Forward: 5'-CTCGCCTTAGTCGTCACCC-3'

Reverse: 5'-CGCCGAAGTTGCATTCCAG-3'

SAV1

Forward: 5'- AAGGCCCAATACAGGCATCC -3'

Reverse: 5'- TGGTCATATTTCACAGGGGCT -3'

SURVIVIN

Forward: 5'- ATGACGACCCCATAGAGGAAC -3'

Reverse: 5'- CGCACTTTCTCCGCAGTTTC -3'

MST1

Forward: 5'- GGAGACGGTACAGCTGAGGA -3'

Reverse: 5'- GCTGCCATAGGACCCTTCTC-3'

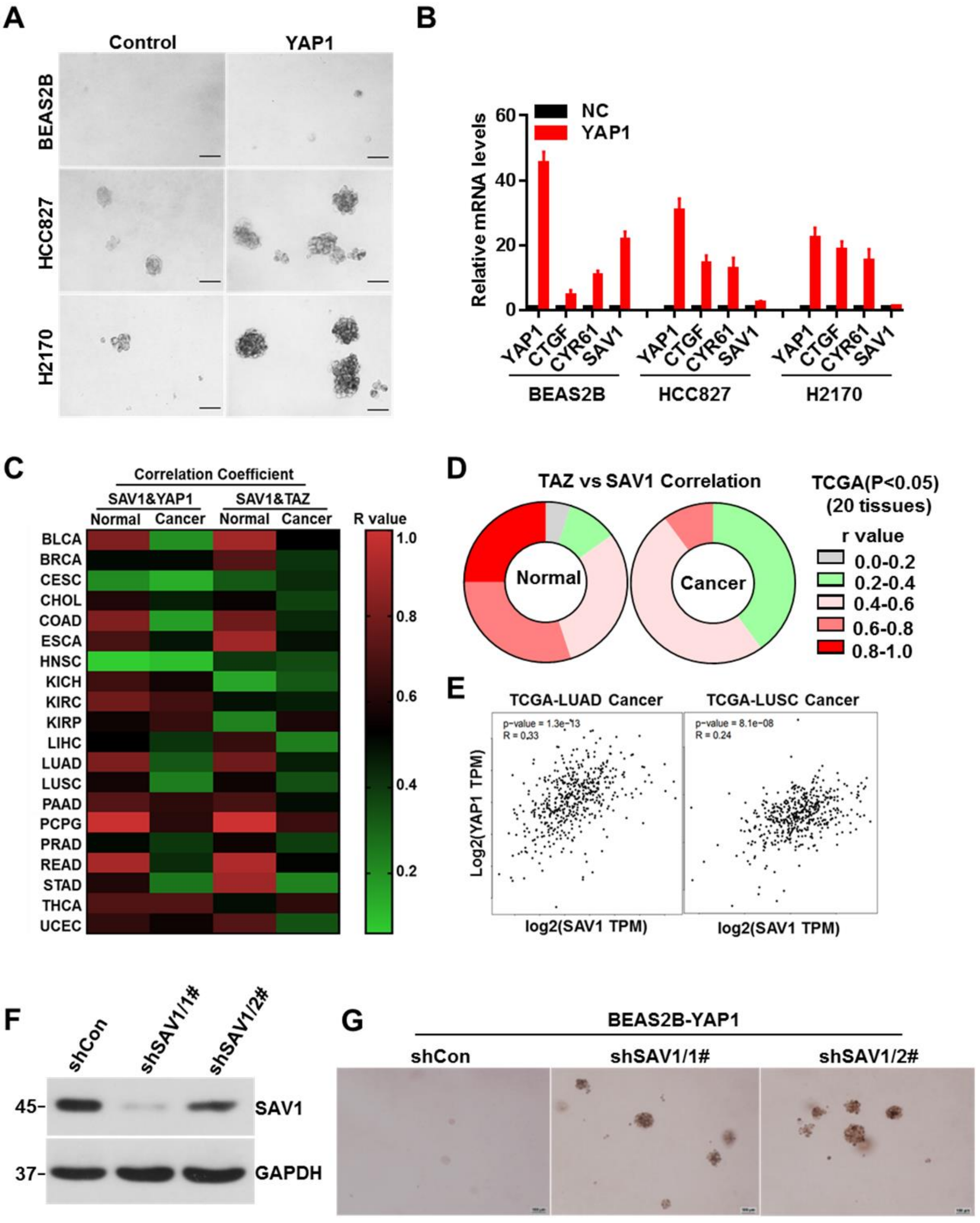
A
Control
YAP1
BEAS2B
HCC827
H2170
B
NC
YAP1
Relative mRNA levels
60
40
20
0
YAP1
CTGF
CYR61
SAV1
BEAS2B
HCC827
H2170
C
Correlation Coefficient
SAV1&YAP1
SAV1&TAZ
Normal
Cancer
R value
BLCA
BRCA
CESC
CHOL
COAD
ESCA
HNSC
KICH
KIRC
KIRP
LIHC
LUAD
LUSC
PAAD
PCPG
PRAD
READ
STAD
THCA
UCEC
1.0
0.8
0.6
0.4
0.2
D
TAZ vs SAV1 Correlation
Normal
Cancer
TCGA(P<0.05)
(20 tissues)
r value
0.0-0.2
0.2-0.4
0.4-0.6
0.6-0.8
0.8-1.0
E
TCGA-LUAD Cancer
TCGA-LUSC Cancer
p-value = 1.3e-13
R = 0.33
p-value = 8.1e-08
R = 0.24
Log2(YAP1 TPM)
log2(SAV1 TPM)
F
shCon
shSAV1/1#
shSAV1/2#
45-
37-
SAV1
GAPDH
G
BEAS2B-YAP1
shCon
shSAV1/1#
shSAV1/2#

Figure.S1

Figure S1, related to Figure 1. YAP-SAV1 negative feedback loop maintain lung epithelial cell

(A) Representative pictures of colony in soft agar. Scale bar: 100 μm. **(B)** YAP, CTGF, CYR61 and SAV1 mRNA levels in different lung cell lines were detected by qRT-PCR. Student's t-test, Mean±s.d. (n=3), **** $p < 0.0001$. **(C)**The correlation between SAV1 and YAP1/TAZ in normal group or cancer group. **(D)** TCGA data shows the stronger correlation between SAV1 and TAZ in normal tissue than cancer group in 20 tissues. **(E)** Using TCGA transcriptome data, the transcripts per million (TPM) expression value shows the positive correlation between SAV1 and YAP in LUAD and LUSC. **(F)** The knockdown efficiency of shRNA was tested by WB assay. **(G)** Representative pictures of colony in soft agar. Scale bar: 100 μm.

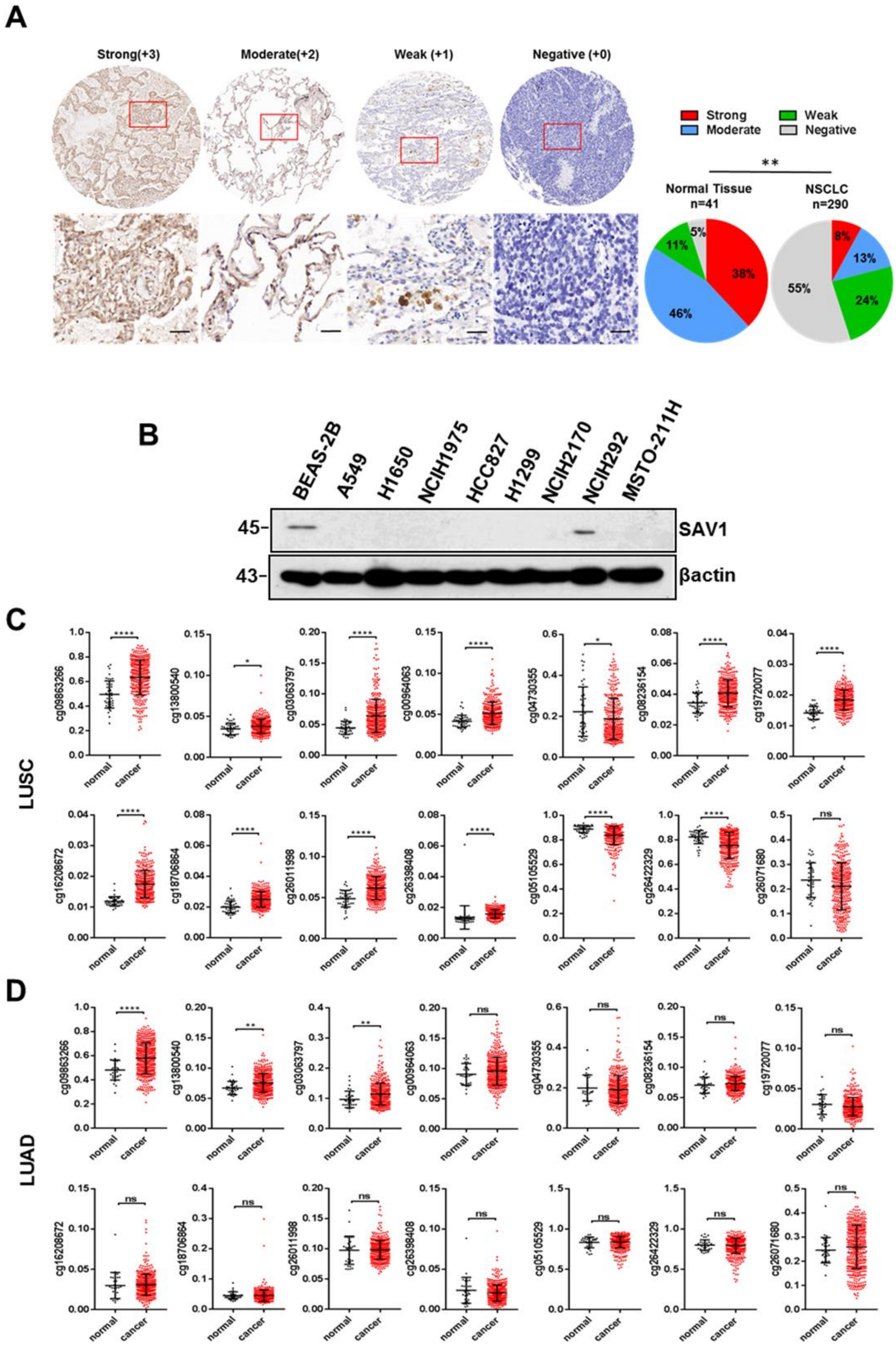
A
Strong(+3)
Moderate(+2)
Weak (+1)
Negative (+0)
Strong
Weak
Moderate
Negative
**
Normal Tissue n=41
NSCLC n=290
38%
46%
11%
5%
8%
13%
24%
55%
B
BEAS-2B
A549
H1650
NCIH1975
HCC827
H1299
NCIH2170
NCIH292
MSTO-211H
45
43
SAV1
βactin
C
LUSC
D
LUAD
cg09863266
cg13800540
cg03063797
cg00964063
cg04730355
cg08236154
cg19720077
cg16208672
cg18706864
cg26011998
cg26398408
cg05105529
cg26422329
cg26071680
normal
cancer
****
*
**
ns

Figure.S2

Figure S2, related to Figure 2. SAV1 promoter methylation inhibits YAP-SAV1 negative feedback regulation in lung cancer cells.

(A) Immunohistochemistry (IHC) was used to determine the level of SAV1 expression in clinical lung tissues. Scale bar: 50 μm. **(B)** WB was used for detecting SAV1 protein expressions in different lung cell lines. **(C, D)** Analysis of the genome-wide DNA methylation data of LUAD and LUSC samples obtained from TCGA dataset (http://cancergenome.nih.gov/) found that a total of 14 cg sites are on the SAV1 gene. Student's t-test, Mean±s.d. **** $p < 0.0001$.

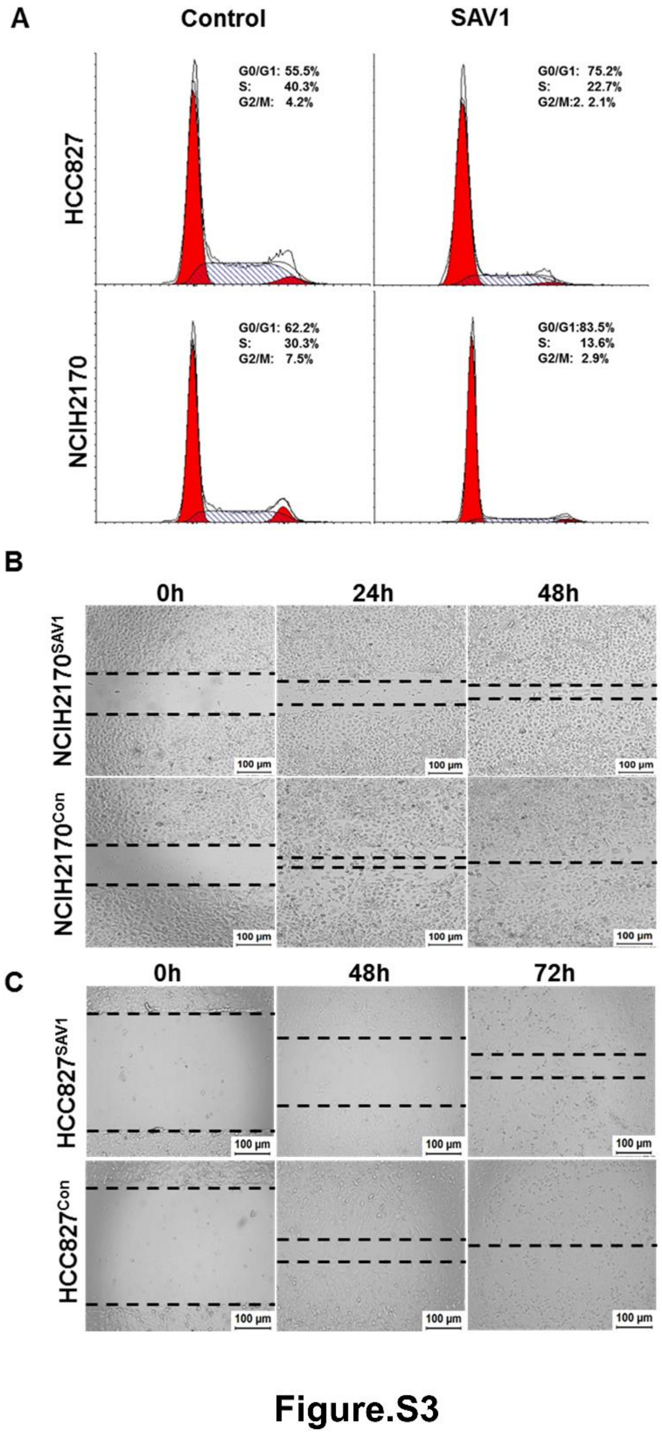

Figure S3, related to Figure 4. Exogenous expression of SAV1 inhibits the malignancy of Lung Cancer Cell.

(A) Cell-cycle distribution analysis was performed by flow cytometry. **(B, C)** To measure cell migration, in vitro scratch wound assay was performed. Scale bar: 100 μm.

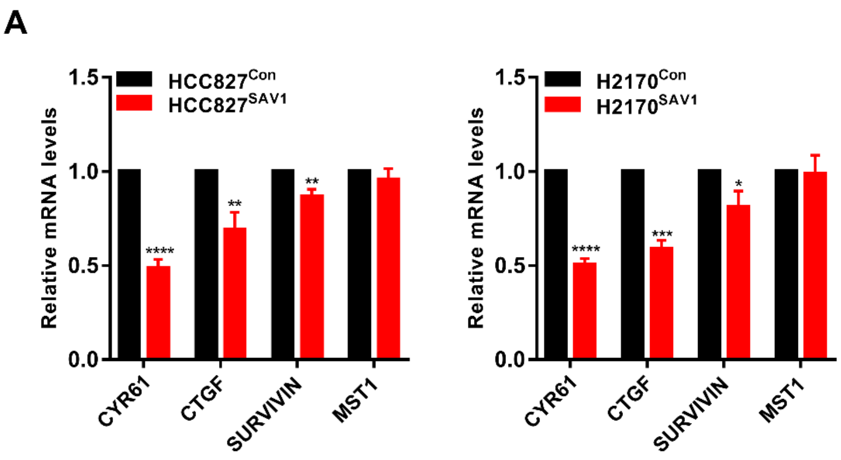

Figure.S4

Figure S4, related to Figure 5. Exogenous expression of SAV1 inhibits YAP transcriptional activity by stabilizing MST1 protein. (A) qPCR was peformed to measure the expression of YAP target genes in HCC827 and H2170 cell lines with or without SAV1 overexpression.
